# Defining Immunogenic and Radioimmunogenic Tumors

**DOI:** 10.3389/fonc.2021.667075

**Published:** 2021-03-19

**Authors:** Terry R. Medler, Tiffany C. Blair, Marka R. Crittenden, Michael J. Gough

**Affiliations:** ^1^Earle A. Chiles Research Institute, Providence Cancer Institute, Providence Portland Medical Center, Portland, OR, United States; ^2^Molecular Microbiology and Immunology, OHSU, Portland, OR, United States; ^3^The Oregon Clinic, Portland, OR, United States

**Keywords:** tumor, T cell, immunogenic, radiation, dendritic cell, priming, immunotherapy, immunogenicity

## Abstract

In the cancer literature tumors are inconsistently labeled as ‘immunogenic’, and experimental results are occasionally dismissed since they are only tested in known ‘responsive’ tumor models. The definition of immunogenicity has moved from its classical definition based on the rejection of secondary tumors to a more nebulous definition based on immune infiltrates and response to immunotherapy interventions. This review discusses the basis behind tumor immunogenicity and the variation between tumor models, then moves to discuss how these principles apply to the response to radiation therapy. In this way we can identify radioimmunogenic tumor models that are particularly responsive to immunotherapy only when combined with radiation, and identify the interventions that can convert unresponsive tumors so that they can also respond to these treatments.

## Introduction—Is My Tumor Immunogenic?

Betteridge’s law of headlines states that if the title poses a question, the answer is “no”. So, this review starts with the proposition that if you have a tumor, it is not immunogenic. It is reasonable to think that years of immunoediting and cancer evolution (1) in the presence of a functional immune system will result in a tumor that is at baseline resistant to immune mechanisms. To help classify tumors and identify appropriate treatments, it is worthwhile to answer two questions: 1. What makes a tumor develop an immune response in the first place?; 2. What determines cancer cell resistance to immune control? Cancer cell resistance to immune control is a highly reviewed topic that focuses on critical immunoregulatory mechanisms such as relative proportions of suppressive T regulatory cells and macrophages, or cancer intrinsic features such as PDL1 expression and antigen processing and presentation. This review will focus on the first question and consider elements of the cancer cells and the tumor environment that determine why some tumors are immunogenic at presentation, which has enormous impact on the choice of treatments and whether they are likely to work. This is much more than a semantic issue of whether an investigator or paper reviewer gets to describe a cell line as immunogenic – there must be some shared absolute measure of immunogenicity that allows us to compare tumor models, identify effective treatments, and extrapolate these data to patients.

**Figure 1:**
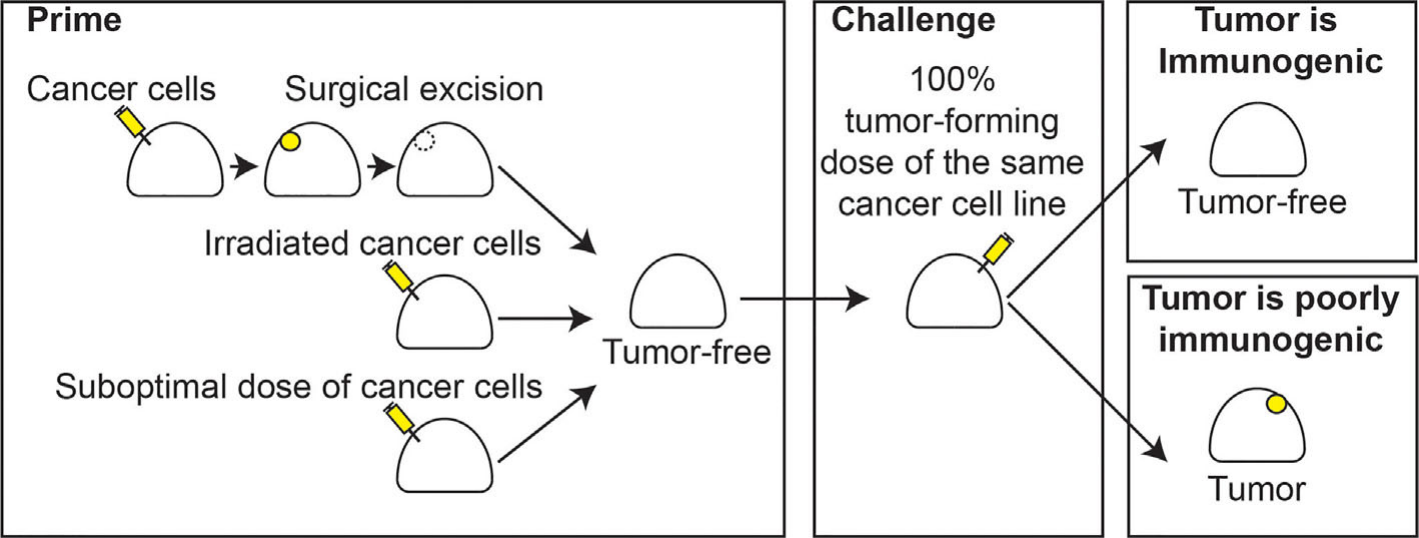
Classic immunogenicity. Classical models of immunogenicity involve a priming step with either injection of a bolus of cancer cells followed by complete surgical resection, injection of irradiated cancer cells, or a suboptimal number of cancer cells that fails to induce tumor formation (left), leaving a tumor-free animal. A challenge step follows, whereby an optimal dose of cancer cells, which would otherwise result in 100% tumor formation in naïve animals, is injected into the animal (center). The animal is followed and if the tumor is rejected, the tumor cells are immunogenic (right, top). If a tumor forms, the cells are poorly immunogenic (right, bottom).

To suggest that tumors that present in patients are not immunogenic is a strong statement that goes against the data from patients treated with currently approved immunotherapies. For example, PDL1/PD1 blocking agents can cure some patients of their tumors purely by blocking a single molecular interaction restraining T cell function. Surely these patients’ tumors are immunogenic. This raises the issue of how we assess immunogenicity. The classic method comes from murine models, where mice are given a first tumor exposure, whether vaccinated with irradiated cancer cells, given a sublethal dose, or given a lethal dose followed by surgical resection, and then the mice are evaluated for their ability to reject a subsequent challenge with a normally lethal dose of the same tumor (2–5). If the tumor does not grow on the second tumor challenge, then it is immunogenic (**Figure 1**). If the first exposure does not cause rejection of the second challenge, it is not immunogenic. Obviously, this measure of immunogenicity cannot be assessed in patients. As we will discuss, this classic model of immunogenicity does not break down the mechanisms of immune rejection, which may result from a failure to sufficiently vaccinate, being resistant to effector destruction, or some combination of both.

## Antigenicity and Immunogenicity

**Figure 2:**
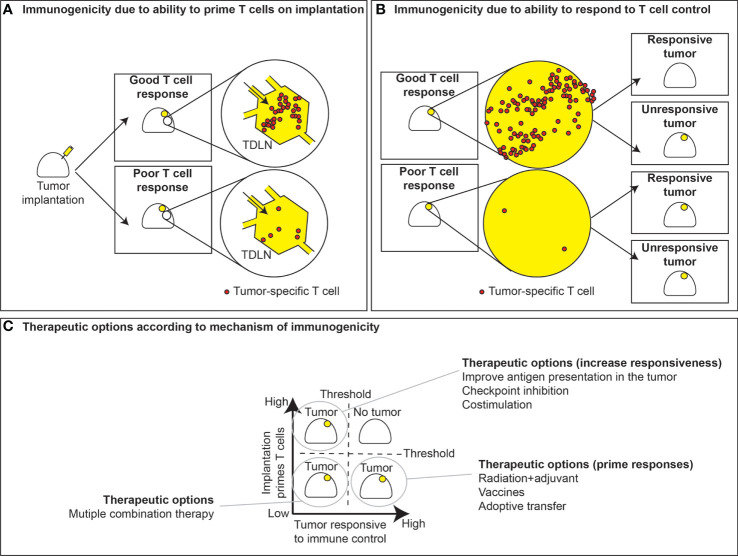
T cell priming versus responsiveness. **(A)** Immunogenic tumors with sufficient antigens and priming elicit good T cell responses in the tumor draining lymph node, while poorly immunogenic tumors fail to generate T cell responses. The ability of tumors to respond to T cell control is not necessarily linked to their ability to prime T cell responses. **(B)** In instances where priming occurs, tumors can either respond to tumor control or fail to respond. Conversely, tumors can either be responsive or unresponsive to T cell control, despite a lack of T cell priming. **(C)** This dichotomy leads to strategies for therapeutic interventions based of whether T cell priming occurs and whether tumors are responsive to immune control. In the case where priming fails yet tumors are prone to immune control, effective strategies may include vaccines or radiation to boost priming or instead ex vivo expansion and adoptive transfer of tumor-specific T cells. Alternatively, in tumors where T cells are primed but fail to exert immune control, therapeutic options may include checkpoint inhibitors, costimulation, or therapies that may improve immune recognition. Instances where both priming and responsiveness are low, tumors may require multiple therapeutic modalities to improve outcomes.

In murine models it has long been known that there is a difference between induced and spontaneous tumors. In mice, tumors formed by highly mutagenic agents such as MCA, or oncogenic viruses that leave viral oncoproteins, are immunogenic as measured by their ability to protect against secondary tumor challenge (3, 4). Tumors that occurred spontaneously in mice (sporadic tumors that lead to classic cell lines such as B16 and 4T1) were not immunogenic – as in they did not protect against rechallenge (2, 3). This mutagenized origin of immunogenic tumors points to antigenicity as a requirement for classic immunogenicity. In agreement with this concept, classic studies showed that treatment of spontaneously derived cancer cells with a mutagen *ex vivo* generated variants that were able to protect against rechallenge (6–9). Importantly, this could include protection against challenge by the parental un-mutagenized strain (6–9). This suggests that in these cases the lack of rejection of the original strain by the immune system was not due to an inability for the cancer cells to be killed, since these tumors can readily be rejected with appropriate vaccination. Rather, these cells fail to elicit sufficiently effective T cell responses on vaccination without the additional supporting antigens (**Figure 2A**). These studies led to multiple investigative approaches testing modifications to the cancer cells that can render a poorly immunogenic tumor immunogenic, purely acting on the priming side of immune responses. For example, the B16 cell line and its multiple variant subclones are poorly protective against rechallenge, but strategies that make them a better vaccine, such as fusion or loading to DCs (10, 11), transfection with cytokines (12, 13), the addition of adjuvants (14), or similar approaches, allows them to protect against rechallenge with the parental clone. Thus, where T cells can be generated, B16 tumors can readily be controlled. Similarly, B16 can be controlled with as few as 10^4^ infused tumor-specific CD8 T cells (15), and where B16 tumor implantation does not generate sufficient T cells to control tumor growth, expansion of these cells *ex vivo* followed by adoptive transfer is protective (16). Since by this definition an untreated, growing B16 tumor does not have sufficient T cells to result in its control, it should not be susceptible to treatments that require these T cells. For example, checkpoint inhibitors such as anti-PD1 require existing suppressed T cells to cure the tumor that can be derepressed with PD1-PDL1 blockade. In support of these data, B16 tumors are resistant to checkpoint blockade, but become susceptible following tumor-specific vaccination of tumor-bearing mice (17, 18). In this way, the B16 model nicely shows the difference between generating an initial anti-tumor immune response, and being susceptible to immune control.

These data suggest that non-immunogenic tumors are deficient in T cells needed for tumor cure. In support of this, Lechner et al. demonstrated that three immunogenic tumors exhibited more T cells in the tumor than three poorly immunogenic tumors (19). However, since the tumor is still growing in mice, these data imply that immunogenic tumors are able to grow despite extensive T cell infiltrates, and thus must have additional resistance mechanisms (**Figure 2B**). In some cases, this is simply the presence of immune checkpoint molecules inhibiting local immunity. In agreement with this, these studies showed that the T cell rich tumors could be controlled by treatment with checkpoint inhibitors, while the poorly immunogenic tumors that lacked T cells at baseline could not be controlled by the same treatment (19). Alternatively, poorly immunogenic tumors may make inappropriate T cell responses that are incapable of controlling the tumor. For example, tumor antigen-specific T cells found in the draining lymph node of growing B16 tumors develop amongst Th2-type cytokine responses, and are incapable of effective anti-tumor immunity (20). Such Th2 cells may drive further immune suppression on exposure to antigen in the tumor environment *via* effects on myeloid cells (21), contributing to tumor progression (22). Thus, in some models, the quality of response when antitumor immunity is initiated can be highly impactful to whether the tumor is considered immunogenic or poorly immunogenic.

According to the criteria discussed above, we can start fitting cancer cells into their categories. For example, B16 is poorly immunogenic because it fails to generate an effective T cell response, though it remains responsive to T cells. MC38 immunogenic since it generates a T cell response, but those T cells cannot kill the tumor without additional intervention so it is relatively unresponsive. However, as you follow the spectrum of immunogenicity to its logical conclusion, there are the tumors that do not grow at all, or are rapidly rejected in immune competent mice. An example of this are tumors generated by MCA mutagenesis in immunodeficient mice (23). These tumors did not evolve under immune pressure and so are spontaneously rejected on injection into immune competent animals (23). Of course, on the other end of the spectrum, it should possible for a tumor to both fail to generate T cells and be resistant to T cells should they be provided. Together, these criteria generate 4 immunogenicity classes of tumors in mice (**Figure 2C**).

In this model, the difference between an immunogenic tumor and a poorly immunogenic tumor is that the immunogenic tumor generates T cells on implantation, but still grows. However, it should be noted that the immunogenicity of tumor injection into mice compared to the growth rate of that tumor is a tunable phenomenon. For example, one of the most important things to know when working with a new tumor cell line is the minimal dose needed for 100% tumor growth. This varies significantly between cell lines, and even a poorly immunogenic tumor can fail to grow if you give too few cancer cells, and a highly immunogenic tumor can grow even in immune competent mice if you give sufficient numbers of cancer cells. The rejection of the primary tumor has long been known to impact immune control secondary challenge with the same tumor line (24), since at the time of secondary challenge the animal will have a larger pool of tumor-specific T cells that may tip the balance towards rejection. This can explain why a prior exposure to immunogenic tumors such as MC38 can help cure a rechallenge with the same tumor, but the primary tumor still grew. At rechallenge, the small number of injected cancer cells can be readily rejected, while a large established tumor can have many log fold larger numbers of cancer cells and also an established, suppressive tumor environment.

For those tumors that fail to make strong T cell responses, we have no information with which we can classify their responsiveness to T cell control. A tumor could be classified as poorly immunogenic based on its inability to protect against rechallenge, but might remain resistant even if tumor-specific T cells were provided by vaccination or adoptive transfer. This means that it would be incorrect to predict that poorly immunogenic tumors merely need a large dose of T cells. Since it would be difficult to distinguish between a poorly immunogenic tumor and an unresponsive tumor until you attempt treatment, this model may need some refinement to be useful to classify tumors. However, it does fit well with more common practical assessments of tumor immunogenicity. Today’s tumor immunologist is less likely to discuss protection against rechallenge, and instead discuss the extent of T cell infiltration or general responsiveness to immunotherapies. These provide a measurable and translationally relevant assessment of the tumor – either tumors are responsive to treatments, or they are not. As each lab works with their favorite tumor models over years of research, investigators come to understand their own models; however, since there isn’t a consistent standard and not everyone attempts the same treatments, this can lead to discrepancies between labs. So, one lab may classify a B16 tumor as immunogenic because it responds to T cell adoptive transfer, but another lab may classify it as poorly immunogenic because it fails to respond to checkpoint blockade. This makes classifying tumors as immunogenic based on a functional response difficult for inter-lab comparisons.

Assessing immunogenicity based on the functional response of a tumor to immunotherapy also generally makes a direct link between the baseline tumor environment and the tumor’s responsiveness to immunotherapy. This fits existing data, since the degree of T cell infiltration is correlative with outcome following checkpoint inhibition in preclinical models (19) and in patients (25, 26). However, the cutoff is ambiguous, as some patients with poorly infiltrated tumors can respond to treatment, and some with highly infiltrated tumors can be unresponsive. For this reason, studies are ongoing by many labs to determine whether there are features of the tumor that predict their sensitivity or lack of sensitivity to specific immunotherapies, and the results of these analyses have clear clinical impact for the use of these therapies. One such effort employs patient-derived organoids, which are emerging as a tool to assess whether patients will respond to checkpoint inhibitors (27). Because they retain both myeloid and lymphoid populations, they can serve as a readout of whether antigen specific T cells are preexisting within a tumor and whether they can be derepressed by immunotherapy (28, 29). Importantly, there is evidence that mechanistic insights gleaned from patient-derived organoids similarly occurs in subsets of patients treated with anti-PD-1 therapy (29). While these models may also be used to identify resistance mechanism and possible combinatorial strategies, caution must be used in interpreting results given the lack of recirculation and the inability to evaluate the ability to prime new responses in such systems.

## Mutational Load, Antigenicity, and Response to Treatment

As discussed above, tumors generated by application of mutagens were shown to be more immunogenic than tumors of spontaneous origin, and that treatment of spontaneously-derived cancer cells with a mutagen *ex vivo* generated variants that were able to protect against rechallenge (6–9). These data suggest that the mutational load in the cancer cells is a critical feature of immunogenicity. In the past decade the ability to identify antigens has changed rapidly with the advances in whole genome sequencing and large-scale bioinformatic identification of neoantigens (30). By modeling the MHC binding properties of predicted mutated neoantigens there is evidence of fewer than expected mutations that are MHC-binding (31), suggesting that there is ongoing immunoediting during tumor formation. Analyses of the overall mutational burden in patients has revealed that a higher mutational burden is associated with an improved outcome in patients treated with PD1 or CTLA4 blockade (32). However, these patients may respond better to any intervention. Hugo et al. demonstrated that while a high mutational burden does not predict outcome to immunotherapies in their analyses, it is associated with increased overall survival regardless of treatment (33). However, there is not a direct link between the mutational burden of tumors and their infiltration with immune cells (34), one of the key features of immunogenicity. Rather than a single factor, mutational burden is best considered along with a range of other tumor-associated features including epithelial-mesenchymal transition phenotypic shifts and other patterns of cancer gene expression that impact the tumor environment (33). In addition, rather than an absolute number of mutations, it has recently become appreciated that a ‘mutator phenotype’ associated with loss of mismatch repair pathways is a stronger predictor of outcome than quantity of mutations (32, 35). *Ex vivo* damage of the mismatch repair pathways can generate tumors that acquire increased mutational burdens (36), and importantly this generates tumors with an impaired ability to grow in immune competent mice but unchanged growth in immune deficient mice (36). Thus, tumors with a high mutator phenotype are classically highly immunogenic.

The different impact of mutations versus a mutator phenotype are important, since not all mutations are equivalent. A recent study demonstrated adding non-clonal mutations can *limit* immune control of primary tumor growth (37). In these studies, despite adding more neoantigens through UV radiation, the more heterogenous the tumor the more likely it was that the tumor would escape immune control. This didn’t result from outgrowth of resistant variants (37). Rather, failure of control related more to an inefficiency of tumor-specific T cells in controlling tumors where not all cells shared the same antigens. Such non-clonal mutations have also been observed in patients and is termed intratumor heterogeneity (38). Detecting non-clonal mutations represents a problem for standard bioinformatic pipelines, since the majority of the cancer cells along with normal cells will be showing an unmutated base, meaning that the minority of sequenced transcripts will read a mutation, so that the mutation is difficult to distinguish from sequencing artifacts (39). By considering the intratumoral heterozygosity as well as the neoantigen burden there is an improved ability to identify patients with longer overall survival (40), and also patients with improved response to PD1 blockade (40). These data suggest that immune responses that can control tumors are most effective when the antigens are shared between all targets, which in turn suggests a role for immunodominant antigens in effective anti-tumor immunity.

Immunodominance is most clearly described in antibody responses, where despite multiple antigenic targets in a foreign antigen, the combination of competitive clonal expansion and ongoing affinity maturation results in a dominance of antibody responses to a small proportion of the range of potential epitopes (41). Affinity maturation does not occur in T cells, but it is common for T cell responses to dominantly focus on specific epitopes in a heterogenous mix despite a range of potential MHC and peptide combinations (42). This is due in part to the finding that only about 1% of potential peptides binds a class I MHC with sufficient affinity to elicit endoplasmic reticulum export and presentation (43). This is particularly evident in influenza, where humans with HLA-B27 generate a T cell response to influenza that is dominated by T cells specific for the influenza nucleoprotein NP383–391, and providing this HLA to mice results in immunodominance to the same peptide (44). In antiviral immunity, immunodominance can present a problem for control of viruses that can alter their target antigens through a high degree of variability (41). Similarly, it is reasonable to think that an immunodominant response to a T cell neoantigen target could present problems if that antigen is not widely shared.

The existence of immunodominance suggests that the initial immune responses to an implanted tumor in preclinical models may result in similar patterns of T cell responses in different mice and even across tumor types. This has been observed to occur in a number of cancers, including MC38 colon adenocarcinoma, B16 melanoma, and MCA-205 fibrosarcoma, where an immunodominant epitope has been identified targeting the endogenous C57BL murine leukemia virus (45). Expression of viral proteins is repressed in normal tissues but has been derepressed in these and other cancer cell lines (45–47). The envelope p15E region is efficiently presented on H2K^b^ and recognized by CD8 T cells. Expansion and adoptive transfer of these antigen specific cells conferred protection against tumor growth and reduced metastasis across multiple tumor types (45). While a major caveat in these experiments is that the mice are genetically identical and share MHC, importantly an alternative viral envelope acts as an immunodominant antigen in tumors such as CT26 (47) and 4T1 (48) in BALB/c mice that have a different MHC haplotype, *via* the AH1 epitope. It is reasonable to think that even in humans, immunodominance towards select targets may still occur despite the diverse neoantigen peptide-HLA combinations. There is practical evidence for this in patients, where investigators have only successfully expanded a small diversity of T cells in each patient – typically T cells specific for 1-3 neoantigens – out of tens to hundreds of identified neoantigen targets in patient tumors (49, 50). One of the reasons why limited numbers of T cell specificities are identified in tumors is technical, relating to the isolation procedure that relies on their ability to grow out of tumor fragments cultured with IL-2. More T cell specificities can be identified by isolating T cells based on expression of activation markers prior to culture (51), which removes competing non-specific or less specific T cells. However, prior to treatment only a small proportion of the potential T cells specific for neoantigen targets can be detected in tumors (52), and in some cases 11 TCR sequences accounted for 90-99% of the tumor specificity (53). The limited diversity of T cell specificities for antigenic tumors presents a problem for therapy. The reliance on individual specificities can result in a huge selective pressure for antigen loss or antigen-presentation loss variants – as has been seen following monoclonal T cell therapy for cancer using highly selected adoptive transfer approaches (49).

The fact that tumors with a mutator phenotype exhibit improved overall survival and response to immunotherapy, yet intratumoral heterozygosity results in the opposite consequence, presents a conundrum. If each cancer cell is capable of accumulating additional mutations *via* its mutator phenotype, each cell should accumulate unique mutations as the tumor progresses – there is no reason for these to be shared. So, it would be expected that a tumor with a mutator phenotype would become increasingly heterologous over time – and so *less* responsive to immunotherapy. Since this doesn’t match with the data, this means much of the mechanism remains to be determined. Importantly, there are indirect mechanisms that may play a role. For example, tumors with the mutator phenotype exhibit increased expression of a range of biomarkers of response to immunotherapy, including increased PDL1 expression (32, 54), and broadly the increased T cell infiltrate in these tumors is counterbalanced by evidence of multiple negative regulatory pathways in the tumor (55). Together these data suggest that immune responses have been generated to antigens in the tumors, but these responses are not curing the tumors. This would be an immunogenic, but unresponsive tumor and it makes sense that the tumor would be responsive to checkpoint inhibition to improve responses. However, this doesn’t explain why the mutator phenotype is correlated with increased mutations and increased infiltration (32, 54, 55), while a high mutation burden in general does not correlate with T cell infiltration (34). The presence of a mutator phenotype can shorten the timeline of tumorigenesis (56), potentially resulting in rapid emergence of tumors without significant immunoediting to eliminate highly immune reactive cancer cell clones. However, early mutator phenotype tumors are highly inflamed before exhibiting a high mutational burden (57), suggesting it is some additional feature of the tumor that drives the immune response (58).

The mutational pathway that leads to carcinogenesis can dramatically impact the immune interactions with the resulting tumor. This can result from cancer cell intrinsic regulation of their immune environment, due to oncogene-driven effects. For example, LBK1 mutant lung cancers have poor immune involvement and respond poorly to immunotherapy (59). The mechanism appears to be *via* LBK1 loss resulting in loss of STING expression in cancer cells (60). The resulting cells are unable to sense aberrant cytosolic DNA forms in the cell that would ordinarily activate the cGAS-STING pathway, and the cells therefore do not activate type I IFN pathways, are less visible to immune cells, and are poorly infiltrated (60). STING expression is inconsistent within a tumor type due to this epigenetic regulation (61), and can vary significantly between different tumor types (62). Other dysregulated programs in cancer cells can activate the STING pathway. Recently, chromosome unstable cancers were shown to accumulate micronuclei at a high rate, and these micronuclei activate cGAS-STING DNA sensing pathways (63). Interestingly, in these experiments activation of the STING pathway accelerated the rate of metastases formation, *via* cancer cell intrinsic NFkB signaling. However, the experiments were performed in immune deficient settings (63), so it is likely that the cancer cell intrinsic effects will be offset by immune regulation of the more visible cancer cells that have an activated STING pathway. Nevertheless, this positive selective pressure for STING expression may explain why STING loss is not an oncogenic event in cancers. In addition, since cGAS-mediated formation of STING ligands can impact neighboring cells *via* intercellular transmission of cGAMP (64, 65), or microvesicle transfer of nucleic acids (66), STING loss in the cancer cell may not eliminate STING sensing in the tumor (62).

These data suggest that features of cancer cells resulting from their mutational pathway to tumorigenesis can impact both their biology and that of the developing tumor. This of course makes sense, as we know that implanting two different cancer cell lines into genetically identical mice can result in two very different tumor environments. Clearly the cancer cells dictate the immune environment. Having some ability to predict the response of the tumor to therapy according to its genomics is one of the major goals in targeted therapy, but we currently have few clear predictors for immunotherapy and few approved immunotherapies. Nevertheless, in some circumstances genomic features of the tumor are used to guide the clinical use of checkpoint inhibitors (67). To better understand how the tumor directs the immune environment of the tumor, it is critical to understand how these immune responses first develop.

## How Does a Growing Tumor Generate Endogenous Anti-Tumor Immune Responses?

To understand how a tumor may generate T cells in the first instance, we must explore the mechanisms that control the priming of tumor specific CD8 T cells in immunogenic versus poorly immunogenic tumors. Chen and Mellman propose that as part of the cancer-immunity cycle, T cell priming against tumor antigens requires: 1) tumor antigens to be released; 2) professional antigen presenting cells (APCs) to take up these antigens; and, 3) adjuvants to be released to activate APCs (68). Defects in any of these steps would result in a failure to prime a productive anti-tumor CD8 T cell response.

Naïve T cells require the extensive costimulatory support of a professional APC to generate fully functional memory and effector populations. Moreover, CD8 T cell responses are most efficiently generated *via* coordinated CD4 T cell help (69–71), so MHC-II expressing APCs are critical for a comprehensive T cell response to tumor-associated antigens. Thus, for initial tumor reactive T cell priming to occur, tumor antigens must first be released and become available to professional APCs. Commonly, antigen release is discussed as a part of cancer therapies, such as following chemotherapy or radiation therapy that result in cancer cell death (72); however, this does not explain how immune responses first develop in untreated tumors. The preclinical data on immunogenicity is skewed by the artifact of tumor implantation into mice. The majority of preclinical tumor experiments involve syngeneic murine cancer cell lines implanted in immune competent mice. This event has long been described as an immunological vaccine-like event, resulting in immune responses to the cancer cells in immune competent mice (73–77). The adaptive immune response generated following tumor implantation can be followed over time to map initial populations of tumor-specific CD8 T cells that can engender anti-tumor immunity, and later development of T regulatory cells that suppress anti-tumor immune control by the CD8 T cells (73–77). This pattern of immune response, suppression, and resistance to subsequent tumor challenge can be impacted by the dose of cancer cells implanted into the animal, which can result in immunological tolerance within critical dose ranges (78, 79). The progressive development of the tumor environment can in part be followed in a progressively growing tumor, such that smaller tumors can exhibit a more permissive immune environment with increased infiltrates of CD8 T cells, but larger tumors proportionally decrease CD8 T cell infiltrates and increase infiltrates of suppressive Treg and myeloid cells (80). As discussed above, that tumors still form in these mice despite the adaptive immunity generated on implantation may be dependent on a dominance of suppression over immunity. However, this suppression is generally a local event, since it is very common that a growing primary tumor can engender sufficient systemic anti-tumor immunity that the mice can reject a second tumor challenge even while the primary continues to grow. This rejection of a second tumor by a mouse with an identical growing primary tumor, first described by Ehrlich, is termed concomitant tumor immunity (81, 82). A range of mechanisms have been considered to explain concomitant immunity (83), but the dominant mechanism is now known to be immunological rejection of the second tumor due to responses initiated following injection of the first tumor. The mechanisms resulting in immunity to injected tumors involves dendritic cells (DCs) functioning as professional antigen presenting cells, since tumors that are ordinarily rejected are able to grow when injected into Batf3^-/-^ mice that lack cross-presenting DCs (84). Thus, cancer cell injection into immune competent mice generates an initial CD8 T cell response *via* cross-presenting DCs, which is therefore a critical feature of immunogenicity in murine models.

In patients, or in mouse models of progressive tumorigenesis that occur without cancer cell injection, this effect may be more difficult to observe. Without the initial bolus of cancer cells to provide debris that may serve as a vaccine event, other mechanisms are required to generate immunity. For example, experiments that use surgical implantation of tumor fragments do not result in anti-tumor immune responses that are observed with implantation of tumor cell suspensions (85, 86). In such cases, to generate T cell responses, cancer cells must transfer antigenic material to APCs in another manner. Soluble cancer-associated antigens can be released from tumor cells – for example PSA is secreted from prostate cancer cells and can be a T cell target for immunotherapy, and mesothelin can be released from pancreatic cancer cells and also serves as a T cell target (87, 88). However, the majority of cellular proteins are not secreted, and therefore will require transfer of cellular material for uptake by APCs. Cancer cells have been shown to release exosomes, which can deliver tumor-associated antigens directly to APCs (89–92). Engineering a tumor to express a model tumor antigen in exosomes resulted in increased tumor immunogenicity, with significantly slower tumor growth than matched tumors engineered to secrete the same antigen, and this growth delay was dependent on an intact immune system (91). Tumors with antigens directed to exosomes were also more immunogenic than those with antigens directed to non-secretory components (93), indicating that the subcellular localization of antigens may be a critical feature of immunogenicity or immunodominance of an individual neoantigen. Importantly, redirection of potential antigens to autophagosomes can increase the immunogenicity of the tumor by generating vesiculated particles that are efficiently cross-presented (94, 95), which may provide an option to increase the immunogenicity of tumors where the potential antigens are not generally directed to exosomes.

Normal programmed tumor cell death is an alternative mechanism for antigens to be released and taken up by APCs. Despite generally increased resistance to cell death in cancer cells (96), DNA damage and metabolic stressors can result in cancer cell death and is particularly pronounced as their growth outstrips the supply nutrients in their environment (96, 97). Multiple types of cell death have been described, however, the two most extensively studied forms are apoptosis and necrosis (98). Apoptotic cell death is typically thought of as being immunologically silent (99, 100) as compared to necrotic cell death, which results in the release of inflammatory signals (101, 102). However, recent work has suggested these pathways are more nuanced and depending on the circumstances, both pathways can lead to the release inflammatory signals (103–106). Thus, some tumors might be classified as poorly immunogenic because they are more resistant to natural tumor cell death, resulting in a failure to release adequate tumor antigens for T cell priming. Alternatively, a cancer cell that is proportionally more resistant to apoptosis may still die if the environment is sufficiently toxic, but through non-apoptotic mechanisms (107). A high rate of cell death in a region of the tumor can overwhelm local phagocytic capacity and result in necrosis. Necrotic material includes a range of endogenous adjuvants with varying ability to stimulate immune responses to associated proteins (102). However, in patients, the presence of pathological necrosis in their tumor is generally associated with poor outcomes across a range of malignancies (108–111). There are likely a wide range of conflicting mechanisms at work in a tumor with extensive pathological necrosis, since a high level of cancer cell death is often correlated with a high rate of cancer cell proliferation (112), and necrotic regions are enriched for macrophages (113) that drive biological pathways to repair necrotic damage (114). As has widely been discussed, these macrophages can be associated with a poor prognosis in patients. These data suggest that the most efficient means of antigen transfer to antigen presenting cells is not necessarily related to high rates of cancer cell death, but may depend on the specific mode of cell death and the means of transfer to antigen presenting cells.

Released tumor antigens will ultimately fail to trigger an immune response unless professional APCs are present to take up these antigens. Dendritic cells excel as professional APCs and multiple dendritic cell subsets exist, each with their own specialized function in immunity (115). Thus, in addition to considering the availability of suitable antigens and maturation signals in tumors, the appropriate type of dendritic cell still needs to be localized in the vicinity of these signals to initiate T cell priming. Conventional type I dendritic cells (cDC1s) are particularly potent at priming cytotoxic CD8 T cell responses (116). Importantly, cDC1s are thought to be the primary cell type capable of cross-presenting tumor-associated antigens to CD8 T cells (117–121). As mentioned above, mice entirely lacking cross-presenting DCs *via* deletion of the cDC1-specific transcription factor Batf3^-/-^ fail to develop anti-tumor T cell responses and even highly immunogenic tumors that are ordinarily rejected can grow in these mice (84). Increased cDC1 signatures in patient tumors correlates with improved survival (119, 122, 123). Moreover, in tumors with very few cDC1s at baseline, administration of drugs that expand cDC1 numbers in the tumor results in improved responses to therapy in murine models (124, 125).

While cDC1s have been shown to have some limited proliferative capacity in peripheral sites, they are typically short-lived and need to be continuously replaced in the tissues by cDC precursors from the blood (126–128). In mice, the chemokine receptors CCR1, CCR5 and CCR6 have been implicated in the recruitment of cDC precursors from blood into tissues, though these requirements likely change during tissue inflammation (129–131). Spranger et al. reported that in their melanoma model, tumor intrinsic β-catenin signaling leads to decreased CCL4 production by tumor cells and impaired recruitment of CCR5-expressing cDC1s into the tumor, ultimately resulting in a failure to prime anti-tumor CD8 T cell responses (132). Alternatively, NK cell-derived XCL1 has also been shown to promote the mobilization of XCR1-expressing cDC1s into tumors and this recruitment is inhibited in tumors that secrete PGE_2_ (122). Tumors implanted into mice that cannot synthesize PGE_2_ are spontaneously rejected, indicating that PGE_2_ is a critical suppressor of immunogenicity in mice (122). These data suggest that different tumors may actively secrete factors that either promote or suppress the recruitment of cDCs to the tumor, and this regulation is highly impactful to classical immunogenicity.

To take up antigens, cDC1s must express receptors that enable them to phagocytose dead or dying cells. These include some of the key markers of the dendritic cell lineage, such as DC-SIGN, CLEC9A, DEC-205 and DCIR (133–137). CLEC9A for example binds to actin filaments that are exposed on dying cells and diverts these antigens be processed in the cross-presentation pathway (135, 138). AXL is another receptor expressed by dendritic cells that is capable of indirectly recognizing apoptotic cells through Gas6 which is bound to phosphatidylserine on the outside of dying cells (139). Moreover, tumor cells themselves have been known to express signals that might prevent them from being recognized and phagocytosed by dendritic cells in the first place, including the “don’t eat me” signal CD47 (140). Elimination of CD47 on tumor cells enhances the development of anti-tumor immune responses in preclinical models *via* dendritic cell-dependent mechanisms (141). Taken together, these data suggest that there are multiple signals that can promote or suppress the uptake of dying cells by dendritic cells and crosstalk between these pathways has important implications for whether or not tumor antigens are taken up by dendritic cells to prime tumor reactive T cell responses.

While many types of material released from dying cells are likely capable of being phagocytosed by APCs, the additional signals released from these cells are critical to determining whether successful priming occurs. Dendritic cells are professional APCs uniquely capable of sensing and integrating signals in their environment to determine whether to initiate an adaptive immune response. In tissues, immature dendritic cells are constantly sampling antigens, but in the absence of maturation signals, productive T cell priming will fail to occur. When dendritic cells receive maturation signals, this leads to a shift from antigen uptake to antigen presentation with increased expression of migratory receptors, cytokines, and T cell co-stimulatory molecules. Naïve T cells lack the receptors for recirculation through tissues, and so must meet dendritic cells presenting tumor-associated antigens in tumor-draining lymph nodes. Dendritic cells also provide T cells with additional co-stimulation and cytokine signals that further support T cell development. During viral or bacterial infection, innate danger signals trigger dendritic cell maturation through pattern recognition receptors such as toll-like receptors (TLRs), C-type lectin receptors or cytosolic nucleic acid sensors. Signaling through these pathways results in the release of type I interferons (IFN) that can further signal back on dendritic cells to promote their maturation. In the absence of infection, dying cells must trigger dendritic cell maturation by releasing endogenous activators of these innate signaling pathways (142). In support of this concept, dendritic cells have been shown to produce type I IFN following tumor implantation in murine models (143). Additional work has demonstrated that when type I IFN is blocked with neutralizing antibodies (144), or instead when dendritic cells lack type I IFN receptors, mice ultimately fail to reject highly immunogenic tumors (145). These data suggest that innate signaling pathways are required for the development of spontaneous tumor reactive T cells.

To understand the nature of the upstream pathways that result in type I IFN release in the absence of infection or therapy it is necessary to study the mechanisms by which nucleic acid sensors are triggered in the tumor environment. Recent work has suggested that following injection of cancer cells into mice, dendritic cells can detect tumor cell derived DNA through stimulator of interferon genes (STING) (146). Woo et al. demonstrated that signaling through the STING pathway resulted in increased expression type I IFN and blocking components this pathway led to diminished tumor specific T cell priming and a failure to reject highly immunogenic tumors (146). It’s also plausible that nucleic acid sensors such as MDA5, RIG-I, or TLR3 function to detect various forms of RNA released by dying tumor cells to trigger interferon pathways. Endogenous retroviral elements are embedded throughout the genome and though their expression is typically silenced, some tumors might be better than others at suppressing the expression of these potentially immunostimulatory RNAs (147, 148). Other potential signals include high mobility group box 1 (HMGB1), a danger signal that has been shown to be released from dying tumor cells that is capable of inducing dendritic cell maturation and tumor regression (149). These data suggest tumors lacking signals that promote dendritic cell maturation may be poorly immunogenic, despite effectively transferring antigen to dendritic cells.

As mentioned earlier, certain tumor-derived metabolites can function to inhibit dendritic cell maturation. Tumors that successfully release antigens and maturation signals, but also secrete factors that inhibit dendritic cell maturation will ultimately result in a failure for these dendritic cells to prime tumor-specific T cell responses. This is illustrated by work from Villablanca et al., which showed that tumors can produce and secrete oxidized cholesterol ligands that bind to the liver X receptor (LXR) and signaling through this pathway in dendritic cells suppresses the expression of CCR7 on maturing dendritic cells (150). As a result, signaling through LXR impaired dendritic cell migration to the LN to prime CD8^+^ T cells, and knockout of LXR in dendritic cells reversed these effects. Other metabolites and signaling pathways that have been shown to suppress dendritic cell function in the tumor, including PGE_2_ as described above and adenosine (122, 151–153). These data suggest that dendritic cell are capable of sensing both activating and inhibitory signals within tumors and the integration of these signals in critical to determining whether a productive anti-tumor immune response is generated. The combination of these mechanisms can determine whether an untreated, growing tumor will have a pre-existing anti-tumor immune response that can be targeted with immunotherapies, or will require additional treatments to initiate anti-tumor immunity.

## Characteristics of a High Surveillance Tumor

As discussed above, alongside the mutational burden the degree of immune infiltrate helps predict whether a patient is responsive to immunotherapy, but these are not necessarily linked (34). There is commonly a coregulated pattern of cell infiltration into tumors, where tumors with a high infiltration of dendritic cells are likely to also have a high infiltrate of T cells and be capable of generating T cell anti-tumor immune responses (132, 154). Since these are potentially overlapping or interrelated mechanisms, it is important to understand what dictates T cell infiltration. One framework outlining the different tumor immune phenotypes is described by Hegde et al. (155) and expanded upon by Chen and Mellman (156). On one end of the spectrum is the immune desert phenotype, largely devoid of T cells in the tumor stroma, with or without infiltrating myeloid cells, that is largely refractory to immune checkpoint blockade. These tumors may have never successfully primed T cells, have deleted the T cells with tumor specificity, or do not recruit T cells into the tumor (155, 157). The second phenotype is an immune excluded tumor, which contains T cells in the tumor periphery or invasive margin, but T cells are absent within other subregions of the tumor, in particular the tumor core. Since tumor-specific T cells are thought to exist in this setting but are restricted to the periphery, the distribution of immune cells in this group of tumors must relate to some difference in recruitment between the different tumor regions. These differences are largely attributed to tumor/stroma interactions, such as a dense fibrotic stroma or vascular features that prevent immune infiltration into the tumor core (155). The third phenotype is the broadly inflamed tumor that has abundant T cell infiltration throughout the tumor, and importantly extends into the tumor parenchyma. Tumors with an inflamed phenotype tend to exhibit type I and type II IFN signatures and respond better to checkpoint inhibitors than those with immune excluded or immune desert phenotypes (158, 159).

Type I and type II signatures characteristic of highly inflamed tumor indicate that efficient cross-presentation by cDC1s has occurred, and retain an ongoing T cell-mediated immune response mechanistically described above. IFNγ signatures are tightly associated with activated lymphocytes, which are the primary source of IFNγ within tumors. CD8^+^ T cells, Th1-type CD4^+^ T cells, γδ T cells, and NK cells are potential sources of IFNγ and are indicative of an immune response against the tumor [reviewed in (160)]. The pleiotropic effects of IFNγ are regulated by cell type-specific expression of IFNγ receptors and their downstream effectors (e.g. JAK2, STAT1, SOCS proteins, IRF proteins, and others) that regulate expression of hundreds of IFNγ responsive genes and cellular behavior. In CD8^+^ T cells, exposure to IFNγ promotes cytotoxic effector functions, motility, and survival (160). In CD4^+^ T cells, paracrine IFNγ signaling reinforces Th1-type responses and actively represses Th2- and Th17-type differentiation (160). Additionally, IFNγ regulates several processes involved in tumor-immune cell interactions, including direct antigen processing and presentation *via* regulation of MHCI, B2M, TAP, and immunoproteasome components (161–164), as well as feedback inhibition of T cell responses *via* the expression of the IFN-regulated molecules PD-L1 and PD-L2 in both tumor and immune cells (165, 166). IFNγ also regulates T cell recruitment *via* regulation of key chemokines (159, 167). Upon exposure to IFNγ, chemokines CXCL9, CXCL10, and CXCL11 are produced by immune cells within the tumor, including macrophages and CD103^+^ DCs (159, 167). This results in chemotaxis into the tumor of activated CD8^+^ T cells that have upregulated CXCR3, the canonical receptor for these ligands (168). CXCR3 is highly expressed on effector CD8^+^ T cells (169) and Th1-differentiated CD4 T cells (170, 171), and their trafficking into tumors is dependent on expression of CXCR3 (168). The importance of CXCR3 and its ligands for CD8^+^ T cell infiltration is underscored by studies revealing CXCR3 and its ligands are prognostic indicators of improved outcome (172–174). Additionally, reduced T cell numbers and worsened outcomes were observed in a subset of ovarian cancer patients in whom CXCL9 and CXCL10 were epigenetically repressed (175). CXCR3 expression on CD8^+^ T cells was recently shown to be repressed by TGFβ, a protein associated with worsened outcomes in patients in colorectal cancer (176). As part of its feedback inhibitory functions, IFNγ also regulates PD-L1 and PD-L2 expression, which negatively regulate CD8^+^ T cell function (165, 166), and at least partially explains anti-PD-1 efficacy in patients bearing an IFNγ signature (177, 178). Together, these data indicate that patients bearing IFNγ signatures, yet still have a growing tumor, have mounted an immune response against their tumor that was subsequently repressed. Logically, de-repression is an appropriate therapy for these patients, and they would therefore be expected to be more responsive to checkpoint therapies.

## Radioimmunogenicity

The effects of radiation therapy on the tumor immune environment have been extensively reviewed. Much of the excitement about the immune component of radiation therapy has been because immune responses provide a large portion of tumor control following radiation therapy in many preclinical tumor models. These data often suggest that the direct effects of cancer cell death initiated by radiation is a minor but essential component of treatment efficacy. While conventional radiation treatment regimens are carefully optimized to ensure cancer cell death while sparing normal cells in the field, it is widely discussed that these regimens could be revisited to optimize their contribution to immune responses (72, 179, 180). In this discussion, as with the discussion of immunogenicity, there is the question of whether radiation is serving as a vaccine event – serving to initiate new immune responses against the tumor or boosting existing immune responses to improve their function – or whether it is assisting effector phase responses to clear residual cancer cells. Certainly, radiation can directly upregulate antigen processing and presentation function in cancer cells, serving to increase their ability to be targeted for effector destruction (181, 182). However, this cannot easily explain the out-of-field effects that have been described in preclinical models and in patients treated with combination therapies (183, 184).

Much of the effort in exploring radiation therapy as a potential endogenous vaccine event have appropriately focused on critical issues in dose, timing, and sequencing of treatments, as well as optimal immunotherapy combinations (180, 185, 186). As the field has developed, it has become clear that as with all other therapies, there are tumor models that are particularly responsive to radiation therapy and radiation therapy combinations. However, studying radiation therapy as a *de novo* endogenous vaccine has been complicated by the *in vitro* model phenomenon of the initial vaccine effect of implanting cancer cells into immune competent mice, as discussed earlier. The initial immune response of implantation means that it is difficult to distinguish a *de novo* effect of radiation from a vaccine boost event (187). Notably, in our studies when we blocked the initial vaccine effect of tumor implantation, radiation therapy was no longer able to combine with immunotherapies for tumor cures (187), even in ordinarily immunogenic tumor models. This is consistent with radiation serving to boost pre-existing T cell responses, but being poorly capable of initiating new immune responses. Importantly, the extent of radiation’s function as an endogenous vaccine is highly model-dependent (188). The importance of radiation therapy as a vaccine event is questioned by studies showing that radiation therapy cannot simply be replaced with strong vaccines (181) – the radiation therapy evidently provides signals that are not present in a vaccine. Similarly, many of the distant tumor therapy models are affected by issues of implantation artifacts. The most common approach used to test distant tumor responses uses implantation of a primary tumor on one flank, and a secondary tumor on the distant flank. Notably, the second tumor is implanted 2-3 days following the primary. This timing avoids the full effect of concomitant immunity that would ordinarily result in rejection and allows the second tumor to grow in the mice. However, the second tumor can develop with a more pronounced immune infiltrate than the primary, different trajectory of immune infiltrates between the tumors (80), and different responses to immunotherapies. This can result in distinct outcomes in the two tumors. For example, in an implanted murine lung carcinoma model, the delayed administration second tumor can respond to systemic anti-OX40 monotherapy with slowed growth, while the primary tumor is not affected (189). Similarly, for highly immunogenic tumors such as MC38, delayed injection of a second tumor can in some cases result in cure of the primary tumor and/or secondary tumor without any additional treatment (190). This cure of the primary wouldn’t happen if a single tumor was injected, so it is possible that since the second injection acts as a vaccine boost event, it increases the overall immunogenicity in the system. While the delayed second tumor injection is a useful tool to readily detect distant tumor effects of primary tumor therapies, it exploits the immune artifact of tumor implantation. It is unclear whether this is relevant to the treatment of metastases, since it is unlikely that in patients metastatic tumors have a more permissive immune environment than the parental tumor.

**Figure 3:**
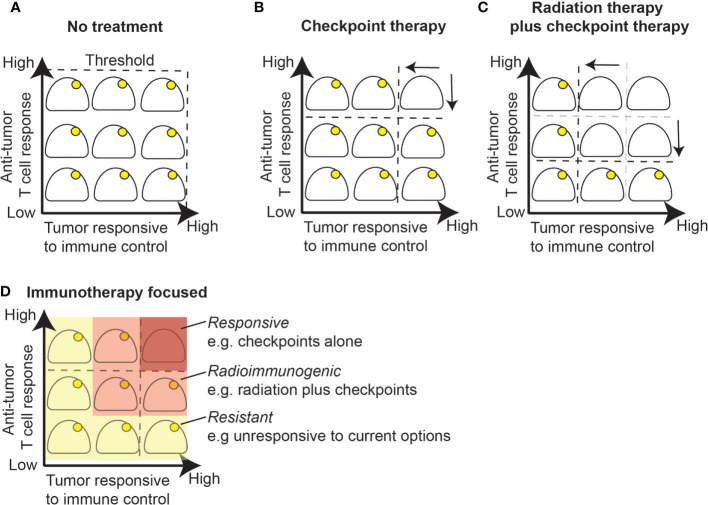
Radiation alters the response threshold to immunotherapy. **(A)** Growing tumors are by definition below the immune control response threshold since a lack of treatment will ultimately be lethal. **(B)** Checkpoint inhibition alone will result in cure for a portion of tumors that have an established T cell response and are responsive to immune control. **(C)** Radiation therapy boosts T cell responses by priming or boosting T cell responses and improves response to immune control within the treatment field due to increased antigen presentation or other inflammatory effects. **(D)** Immunotherapy changes the response threshold while radiation further changes the response threshold in radioimmunogenic tumors. A portion of remaining tumors fail to respond well to checkpoint inhibition with or without radiation therapy and will require additional therapeutic modalities that target additional resistance mechanisms.

Despite this caveat, there are a range of tumor models such as the BALB/c mammary tumor cell line 4T1 that are classically poorly immunogenic and are not treatable with checkpoint inhibitor monotherapy. However, following the combination of radiation therapy with checkpoint inhibitors, both the irradiated tumor and the unirradiated tumor can be controlled (191). This tumor is not immunogenic, so is this tumor radioimmunogenic? The term ‘radioimmunogenic’ is a useful tool to discriminate those tumors that may be treatable by adding radiation therapy to immunotherapy (**Figure 3**). In addition, by comparing such tumors it may help us identify features of the tumor that dictate responsiveness to radiation. For example, in our hands, the Panc02 model of pancreatic adenocarcinoma is unresponsive to any T cell targeted therapy combined with radiation therapy. This includes therapeutic antibodies to targets such as CTLA4, PD1, and OX40, which work very well in other models. However, Panc02-SIY, which has been engineered to express the strong model antigen SIY is responsive to these combinations (181, 187). Thus, while the parental Panc02 cell line was generated by MCA carcinogenesis (192), it appears insufficiently antigenic to be radioimmunogenic. As we have compared tumor models to understand why some tumors respond to radiation and others do not, we identified that the poorly responsive tumors failed to mature DCs in the tumor environment following radiation therapy (188). In radioimmunogenic models such MC38 and MOC1, DCs in the tumor upregulated maturation markers following radiation therapy, showed similar maturation in the tumor draining lymph node, and the eventual tumor control was dependent on trafficking of T cells through the blood and to the tumor (188). In poorly immunogenic tumors such as Panc02 and MOC2, this loop was broken, and T cells were not able to contribute to tumor control following radiation. This could be restored through application of DC-targeted adjuvant to the tumor environment, restoring DC maturation and T cell control of the tumor (188). In radio-immunogenic MC38 tumors, the therapeutic efficacy of radiation has been shown to rely on STING-dependent cytosolic DNA sensing pathways in DCs (193) and reports have suggested that radiation is capable of driving the expression of enzymes in tumor cells that function to degrade potential immunostimulatory DNA signals (194). This suggests that the adjuvant balance in poorly radio-immunogenic tumors following radiation therapy is suboptimal and these result may explain why immunological adjuvants have long been described as effective immunotherapies in combination with radiation therapy (195, 196). Since myeloid cells in the tumor are a critical target for immunological adjuvants (195, 197, 198), this suggests that myeloid cells may be a limiting factor in poorly radioimmunogenic tumors. This fits our experience with the parental Panc02 tumor model, since while it is unresponsive to T cell targeted therapies as discussed above, it has proven responsive to radiation therapy combined with therapies targeting myeloid populations in the tumor environment, including therapies targeting NFkB p50, Mertk, TGFb, and STING (199–201).

Unbiased exploration of features of the tumor immune environment clearly demonstrate that patterns of myeloid infiltrate can correlate with patterns of T cell infiltrate and impact patient outcome. For example as discussed earlier, DC infiltration and CD8 T cell infiltration are commonly correlated (167, 202). This can present a chicken and egg question as to which population causes infiltration of the other, but as discussed above, limiting DC infiltration into tumors also limits T cell infiltration (132), and mice lacking DCs fail to generate tumor infiltrating T cell populations (84). Together these data suggest that in poorly radioimmunogenic tumors the initial biology that results in DC activation and subsequent generation of anti-tumor T cell responses are deficient. However, improving DC responses following radiation therapy using CD73 blockade also improves responses to radiation and anti-CTLA4 in 4T1 tumors (203), so it is likely that DC targeting has the potential to be widely applicable to improve responses in radioimmunogenic tumors.

The effects of radiation therapy and immunotherapy in these models can be seen as altering the threshold of immunogenicity. A growing, established tumor given no further treatment is lethal and so any immune responses are by definition below the threshold to eliminate the tumor **(****Figure 3A****)**. Some tumors may be responsive to checkpoint inhibitor therapy, which means that blocking suppressive mechanisms can permit a substandard immune response to successfully eliminate the tumor **(****Figure 3B****)**. These *responsive* models do not need radiation therapy for tumor control. A second group of tumors do not respond to checkpoint inhibitors alone, but can be cured by radiation therapy combined with checkpoint inhibitors (**Figures 3C, D**). In these *radioimmunogenic* models, the addition of radiation therapy alters the threshold of response. This can occur by priming or boosting T cell responses, by improving effector function within the field due to effects on inflammatory or antigen presentation effects, or some combination of local and systemic effects. A final group of tumors remain *resistant*, where combination therapies remain unable to cure these tumors. If we consider 4T1 tumors, these are classically *poorly immunogenic*, aggressive tumors in mice. However, they are clearly *radioimmunogenic*, since they respond very well to radiation therapy plus anti-CTLA4, and provide an excellent model of local and distant tumor control by experimental immunotherapies.

**Figure 4:**
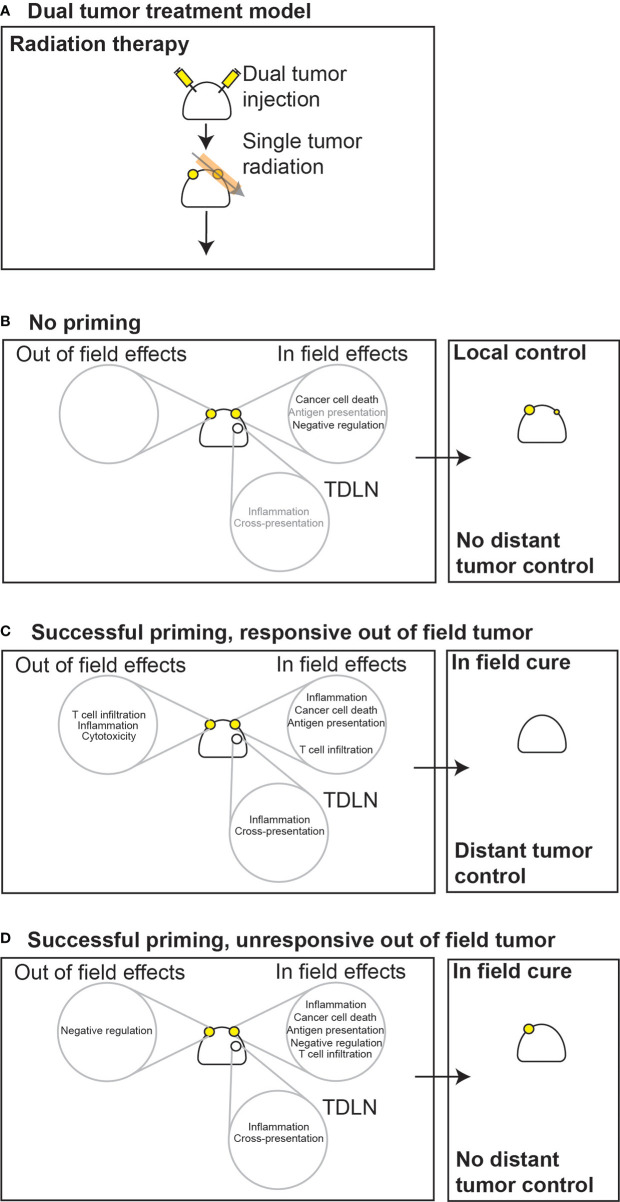
Local and distant responses to radiation therapy or combined radioimmunotherapy in immunogenic or poorly immunogenic tumors. **(A)** In dual treatment models, immune responses initiated in one tumor must be able to affect distant tumors to result in their control. **(B)** In poorly immunogenic tumors that have limited pre-existing immunity, if radiation is unable to prime new immune responses then distant tumors will be unaffected. **(C)** If radiation successfully primes new responses or boosts existing responses, if the distant tumor is responsive to increased numbers of T cells then distant tumor control will be observed. **(D)** If radiation successfully primes new responses or boosts existing responses but the distant tumor is unresponsive to these T cells, then distant tumor control will not be observed. However, since radiation has additional in-field effects on inflammation and antigen presentation, the treated tumor may still be cured through immune mechanisms. Gray lettering, low occurrence; solid black lettering, moderate occurrence; bold black lettering, high occurrence. TDLN, tumor draining lymph node.

This ability to control distant tumors is obviously an extraordinarily important opportunity to impact patients with metastatic disease. In some tumors, radiation therapy is unable to prime new T cell responses in the draining lymph node, so would not be expected to impact a distant tumor outside of the treatment field (**Figures 4A, B**). If radiation therapy successfully primes or boosts T cell responses resulting in increased circulating tumor-specific T cells, then there remain multiple options. If the out-of-field tumor is responsive to T cells, then the distant tumor may be controlled (**Figure 4C****)**. However, tumors that are resistant to effector mechanisms could be unaffected by radiation (**Figure 4D****)**. For example, some tumors already have a good T cell infiltrate, but grow regardless. More T cells may not greatly alter the threshold for these tumors since they already suppress local immunity *via* a range of mechanisms that include PDL1-PD1 or CD80/86-CTLA4 interactions. This can result in differential responses in the in-field versus out-of-field tumors. If radiation is optimal it may result in a range of local effects such as increased inflammation and direct antigen presentation due to nucleic acid sensing (195, 197), and loco-regional effects that include tumor antigen cross presentation in an inflamed draining lymph node (188). While this can result in increased numbers of tumor specific T cells entering the circulation, the effects of radiation therapy on antigen presentation and inflammation will not be taking place out in the out-of-field tumor. This means that while we can optimize the dose and timing of radiation therapy to increase in-field inflammation and T cell priming, these events will not affect a distant, unresponsive tumor. Using these criteria we can start to identify responsive versus unresponsive tumors. If our therapies can impact the distant tumor, they must be responsive to immune therapies that rely purely on increased tumor-specific T cell numbers. A recent example of this can be seen in Ruckert et al., where using a dual flank tumor model, they demonstrated that systemic vaccination against tumor specific antigens only impacted the growth of the irradiated tumor (204). Although the distant tumor was injected a few days following primary tumor injection and therefore had an improved tumor environment, it remained resistant to increased circulating tumor-specific T cells (204). Systemic immunotherapies can impact these thresholds in the distant tumor. As single agents, systemic administration checkpoint therapies can cause an increase in baseline inflammation in tumors by eliminating negative regulation of T cells. This may alter the threshold in a distant unresponsive tumor that allows it to be controlled by T cells, becoming responsive. This agrees with the published data, since tumors that have higher numbers of T cells and increased clonal expansions of T cells are also the most responsive to checkpoint inhibitor therapy (205, 206). However, it is difficult to isolate the effects of these systemic therapies. For example, anti-CTLA4 has been shown to improve T cell responses to tumors associated antigens in the tumor-draining lymph node, the treatment site, and in the distant tumor. This can result in control of both the irradiated and unirradiated tumor. In this case, did anti-CTLA4 function primarily to increase priming, to remove resistance, or is it always some combination of both?

The relative effects of immunotherapies on ‘in field’ versus ‘out of field’ tumors may be clearer where the therapy is focused specifically on one mechanism. For example, in mammary carcinoma models, CD73 blockade increases DC infiltration in the irradiated tumor, but not the non-irradiated tumor (203). While this improved local control, it did not improve control of distant tumors that were established either as spontaneous metastases or *via* dual flank injection. Therefore, the distant tumor remained resistant to T cell responses that were generated by treatment. In the above dual tumor model used by Ruckert et al., the addition of anti-PD1 to radiation therapy improved primary and distant tumor control, but distant tumor control was still not impacted by tumor-specific vaccination (204). Notably, the immunomodulatory effects of radiation including upregulation regulatory molecules such as PDL1 were restricted to the tumor in the radiation field, and did not impact the out of field tumor (204). Therefore, the out of field tumor remained resistant, despite in field success. As another example, in our hands direct injection of STING ligands into Panc02 pancreatic adenocarcinoma tumors in combination with radiation therapy resulted in local tumor cure (199). Using Panc02 tumors expressing the model antigen SIY allowed us to demonstrate that the combination generated more tumor-specific T cells in the circulation, but this had only a moderate effect on the distant tumor and was not observed with either treatment alone (199). However, in B16 tumors expressing SIY, STING ligand administered to a tumor was able to result in systemic tumor-specific T cell responses and caused distant tumor cure as a single agent (207), implying that the distant B16 tumors are highly responsive to T cells once they are generated. In a lung metastases model where STING ligands were delivered to both lungs *via* inhalation but only one region irradiated, the combination with radiation was able to control tumors inside and outside the field (208). Importantly, both the in-field and out-of-field tumor control was dependent on CD8 T cells. Thus, in-field therapies that generate T cells are not necessarily able to control *resistant* out-of-field tumors, unless the out-of-field tumor also receives treatment. When we deliver systemic immunotherapies, such as PD1 or CTLA4 blocking antibodies, it can be difficult to discriminate where these therapies produce their effect.

## Conclusion

According to the above discussion, an immunogenic tumor may have a tumor-specific T cell response, but since it is a growing tumor it will be resistant to these T cells without additional intervention. These tumor-specific T cells will have been generated *via* DC cross-presentation, despite any negative pressures from tumor-infiltrating macrophages, T regulatory cells, or metabolic effects of the tumor. For such tumors, overcoming T cell suppression could be sufficient to result in tumor control, and these tumors also appear more responsive to a range of conventional therapies (209). For poorly immunogenic tumors, the result is less clear. It is possible that the tumor can be manipulated to generate effective T cell responses through treatment, adoptive transfer, or vaccination. However, we still will not know whether the tumor will additionally be resistant to control by the effector phase of immune responses, just like the immunogenic tumor. Therefore, for tumors that present with absent immune infiltrates it is likely that combination therapies will be necessary.

In answer to the question posed at the start of this review “Is my tumor immunogenic?”, one would hope that the answer is “Yes”. In that case, many therapies will work optimally, not just immune therapies. For the remaining patients, there will be some who have tumors that are radioimmunogenic. In these patients, radiation therapy in combination with immunotherapies have the potential to control their tumor. For this to impact patients with metastatic disease, then the out-of-field tumors will also need to be responsive to immunotherapies, since these distant tumors will not receive radiation. For this reason, clinical studies designed to treat metastatic tumors with radiation therapy to a distant site should incorporate approaches that increase the responsiveness of the out-of-field tumor potentially through administration of systemic agents that target the suppressive tumor environment, and not just improve radiation’s ability to act as a vaccine. By contrast, a therapy that aims to increase local control following radiation therapy may not require systemic therapy and can focus on immune mechanisms that assist the radiobiological response to radiation treatment within the field. Therefore, it will be critical to match the trial design to the agent, as well as the agent to the intended outcome (210). In either case, an ability to discriminate immunogenic and radioimmunogenic tumors will help us understand how our preclinical models might apply to specific clinical scenarios. This will help ensure we are developing appropriate therapies for patients, and not just for our artificial preclinical settings. This will better address why radiation plus immunotherapy is overwhelmingly successful in preclinical models, but these do not necessarily result in successes in randomized clinical trials.

## Author Contributions

TM, TB, MC, and MG: writing and editing. All authors contributed to the article and approved the submitted version.

## Funding

This work was supported by NCI R01CA182311, R01CA244142, and R01CA208644. The funders played no role in the content of the manuscript.

## Conflict of Interest

MG and MC received research funding from Bristol Myers-Squibb, Jounce, and Mavupharma that is unrelated to the content of this manuscript.

The remaining authors declare that the research was conducted in the absence of any commercial or financial relationships that could be construed as a potential conflict of interest.
